# Optimization of Ultrasonic-Assisted Extraction of Major Phenolic Compounds from Olive Leaves (*Olea europaea* L.) Using Response Surface Methodology

**DOI:** 10.3390/foods7090149

**Published:** 2018-09-06

**Authors:** Jasminka Giacometti, Gordana Žauhar, Marta Žuvić

**Affiliations:** 1 Department of Biotechnology, University of Rijeka, Radmile Matejčić 2, 51000 Rijeka, Croatia; marta.zuvic@uniri.hr; 2 Department of Medical Physics and Biophysics, Faculty of Medicine, University of Rijeka, Braće Branchetta 20, 51000 Rijeka, Croatia; gordana.zauhar@medri.uniri.hr; 3 Department of Physics, University of Rijeka, Radmile Matejčić 2, 51000 Rijeka, Croatia

**Keywords:** ultrasound-assisted extraction, response surface methodology, olive leaves, oleuropein, verbascoside, luteolin-4′-*O*-glucoside

## Abstract

The ultrasound-assisted extraction (UAE) of oleuropein (OLE), verbascoside (VER), and luteolin-4′-*O*-glucoside (L4OG), as the major phenolics from olive leaves, was optimized using response surface methodology (RSM). A Box–Behnken design (BBD) was used to monitor the effect of different modes of ultrasound operation (pulsed and continuous), liquid–solid (L–S) ratio, and sonication time on each phenolic yield. The yield of UAE and conventional solid extraction (CSE) was determined after performing ultrahigh-performance liquid chromatography with a diode-array detector (UHPLC-DAD) analysis on the extracts. The results suggested that, under optimal conditions, the concentrations of OLE, VER, and L4OG were 13.386, 0.363, and 0.527 mg/g of dry powdered olive leaves (DPOL), respectively. Verification of experiments was carried out under the modified optimal conditions and the relative errors between the predicted and experimental values were dependent on the examined phenolic compound (OLE 8.63%, VER 11.3%, and L4OG 22.48%). In comparison with CSE, UAE improved the yields of OLE, VER, and L4OG (32.6%, 41.8%, and 47.5%, respectively, after 1 min) at a temperature of 60 °C, an L–S ratio of 15 (*v/w*), and in the continuous mode of UAE. We demonstrated that the UAE technique is an efficient method for enhancing yields of OLE, VER, and L4OG in olive-leaf extracts, while the chosen model was adequate to optimize the extraction of major phenolic compounds from olive leaves.

## 1. Introduction

Olives are one of the most produced crops worldwide, especially in the countries of the Mediterranean region. A large amount of generated waste and by-products during the olive-oil production process could be a great source of high-added-value compounds, especially bioactive compounds. Due to numerous possible applications, phenolic compounds isolated from by-products of the olive-oil production process are of top scientific and socioeconomic interest [1].

Until recently, the waste products of olive-tree plantations were commonly disposed of over open land, thus becoming an environmental problem. Therefore, their processing supports the industrially sustainable production of olive-based products [1,2]. From the point of view of the possible exploitation of this agro-waste, it is important to emphasize that the phenolic profile of olive leaves depends on several factors, such as cultivar type and geographical origin, age, moisture content, and the degree of ripeness, as well as the proportion of branches on the tree and the degree of soil contamination [3]. 

**Figure 1:**
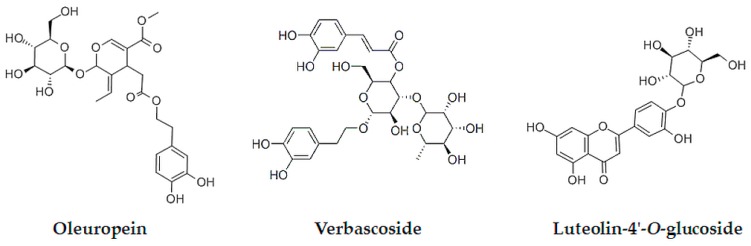
Structures of the most abundant phenolic compounds in olive leaves.

Oleuropein (OLE) is the most abundant phenolic compound present in olive leaves [4,5], followed by verbascoside (VER), luteolin-4′-*O*-glucoside (L4OG), and other phenolic compounds (see Figure 1). The primary role of OLE in the developmental processes of olive trees is related to fruit development and maturation [6]. On the other hand, the highest levels of L4OG and VER were found in the mature leaves [7]. 

Those bioactive compounds display various effects on human health, primarily as antioxidants and activators of gene expression, as well as displaying an effect on healthy gut microbiota and maintaining the efficacy of the mitochondria [8,9,10]. For these reasons, they are used in the production of natural pharmaceutical supplements, as well as additives for the food and cosmetic industries [11].

Compared to conventional extraction methods of bioactive compounds, modern non-conventional extraction methods were developed as a solution to improve conventional extraction by reducing mass transfer limitations and increasing yields in a shorter time, with minimal consumption of extraction solvents. Several novel methods are effective for the extraction of bioactive phenolics, including ultrasound-assisted extraction (UAE), microwave-assisted extraction (MAE), sub- and supercritical fluid extraction (SFE), pressurized liquid extraction (PLE), pulsed electric fields (PEF), and high-voltage electrical discharges (HVED) [12]. However, there are some limitations of innovative technologies such as high investment costs, insufficient monitoring of the variables during processing, and a lack of regulative measures, as well as industrial implementation [13]. In addition to these limitations are also those related to the type of plant material and its characteristic properties.

The use of low-frequency ultrasound sources (20–40 kHz) became increasingly common in the use of sonication for the extraction from plant material. Ultrasonic waves induce pressure and cavitation with disruption of cellular walls by facilitating the release of target components. Solvent selection plays a crucial role in UAE, mainly due to its significant influence on cavitation effectiveness, as well as in acoustic energy transference to reactants [14]. In addition, cavitation effectiveness depends on the frequency and intensity of ultrasound, medium properties (viscosity and surface tension), and ambient conditions (temperature and pressure). Moreover, the ultrasound technique is applicable for pre-treatment or for enhancing extraction efficiency in combination with solvents in order to improve cell membrane permeability [15]. Although the long extraction time in continuous mode is observed to significantly increase the antioxidant yields, a negative effect on antioxidant activity was found compared to pulsed mode [16].

Response surface methodology (RSM) is an efficient mathematical and statistical tool that is widely used to optimize experimental parameters in various processes and to investigate their possible interactions [17,18]. In comparison with single-variable optimization methods, RSM evaluates different interactions and the key experimental parameters widely applied to optimize many processes. RSM is an efficient tool for the optimization of the extraction processes from plant materials. A Box–Behnken design is a spherical design, and it is frequently applied for the RSM optimization of extractions, including UAE [19].

In the present study, RSM was applied to the optimization of UAE parameters such as the pulsed or continuous ultrasonic operating mode, sonication time, and the liquid–solid (L–S) ratio for optimal yields of OLE, VER, and L4OG from dry powdered olive leaves (DPOL). The concentrations of OLE, VER, and L4OG from DPOL after UAE and conventional solid extraction (CSE) were determined using ultrahigh-performance liquid chromatography with a diode-array detector (UHPLC-DAD). The obtained yields of OLE, VER, and L4OG in optimized UAE conditions from olive leaves were compared with CSE.

## 2. Materials and Methods 

### 2.1. Plant Material

Olive-tree leaves were picked randomly from the same tree of the Busa variety grown in Vodnjan (in southwest Istria, Croatia, 44°57′40′′ N–13°51′10′′ E) on 20 November 2014. After being collected, leaves were dried and then stored at ambient temperature without sunlight. Dry olive leaves were milled to form a powder before extraction processes.

### 2.2. Chemicals

Ethanol, formic acid, methanol, and acetonitrile (HPLC grade) were purchased from J.T. Baker (Avantor Performance Materials B.V., Deventer, The Netherlands). Oleuropein (OLE), verbascoside (VER), and luteolin-4′-*O*-glucoside (L4OG) were purchased from Extrasynthese (Genay CEDEX, France). Pure water was obtained from a Millipore Milli-Q water purification system (Merck, Darmstadt, Germany). 

### 2.3. Apparatus and Extraction

UAE was conducted using an ultrasonic homogenizer (Sonopuls model HD 2070, Bandelin, Berlin, Germany) equipped with a titanium-alloy flat-tip probe (13 mm in diameter; VS 70 T, Bandelin, Berlin, Germany) with a frequency of 20 kHz. The amplitude was controlled to set the ultrasonic output power at 70% of the maximum range of both working modes. Pulsed and continuous modes of operation were used for extraction. The pulsed mode was carried out with two and five cycles, while 10 cycles corresponded with the continuous mode of operation. 

Dry powdered olive leaves (DPOL) were added to the fixed volume of 10 mL of previously thermostated 80% aqueous ethanol (at 60 °C) to reach L–S ratios of 15, 20, and 25. The vessel was placed in a thermomixer (Eppendorf, Hamburg, Germany) and UAE was performed. After sonication was completed, the solids were separated from the mixture by centrifugation (Eppendorf 5427R, Hamburg, Germany) for 5 min at 5000 rpm and 4 °C. Each supernatant was filtered through a 0.2-µm nylon syringe filter prior to analysis using UHPLC, in order to monitor the yield of extraction.

Conventional solvent extraction (CSE) was carried out as a control group to compare with UAE. Samples were extracted with 80% aqueous ethanol at 60 °C; the L–S ratio was 15, and extraction times were 1, 2.5, and 5 min. Samples were prepared for UHPLC analysis using the procedure described previously.

#### 2.3.1. Extraction Yield

The yield of the examined phenolic compounds (OLE, VER, and L4OG) was determined by calculation of concentration using UHPLC-DAD in relation to the weight of the plant material used. Accordingly, the concentration of oleuropein was calculated using Equation (1).

(1) $$\;\mathrm{Concentration}\;\mathrm{of}\;\mathrm{Oleuropein}\;\left(\frac{\mathrm{mg}}{g}\right)=\frac{\mathrm{Oleuropein}\;\left(\mathrm{mg}\right)}{\mathrm{Plant}\;\mathrm{sample}\;\left(g\right)},\;$$

where the concentration of oleuropein (OLE) in mg was determined using the UHPLC-DAD method from one extraction trial of a plant sample weight of dry pulverized olive leaves (in g).

The concentrations of verbascoside (VER) and luteolin-4′-*O*-glucoside (L4OG) were calculated using the same procedure. All extraction experiments were carried out in duplicate.

#### 2.3.2. UHPLC-DAD Analysis of Oleuropein, Verbascoside, and Luteolin-4′-*O*-Glucoside 

**Figure 2:**
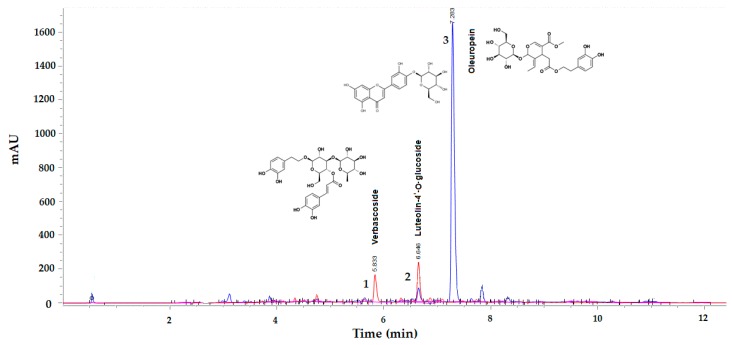
Representative ultrahigh-performance liquid chromatography with a diode-array detector (UHPLC-DAD) chromatogram of the olive-leaf phenolics extract. Peaks 1 and 2 were detected at 325 nm (lines in red), while peak 3 (line in blue) was detected at 280 nm.

The Agilent 1290 Infinity LC system (Agilent Technologies, Palo Alto, CA, USA) equipped with ChemStation software and a diode-array detection (DAD) system was used for olive-leaf-extract analysis [20]. Separations were achieved on a Zorbax RRHD SB-C18 (2.1 mm × 50 mm, 1.8-µm particle size), operated at 30 °C, with a 0.2-µm stainless-steel inline filter. The mobile phase was 0.1% formic acid in water (solvent A) and acetonitrile (solvent B) at a flow rate of 0.3 mL/min. The gradient started with 0% B to reach 5% B at 1.8 min, 15% B at 3.6 min, 40% B at 13.0 min, and 100% B at 14.5 min. The total run time was 17.5 min. Chromatograms were recorded at 280 and 325 nm from the DAD, and data were collected between 200 and 600 nm. The injection volume was 1 µL. A representative chromatogram is presented in Figure 2.

The identification of OLE, VER, and L4OG dissolved in 80% aqueous ethanol was carried out by retention time mapping with set standards. An external calibration method was used for the quantification of OLE, VER, and L4OG in olive-leaf extracts. Briefly, a stock solution of 1 mg/mL of standards (OLE, VER, and L4OG) was dissolved in 80% aqueous ethanol and serially diluted in the same solvent to produce standard solutions of 0.0156, 0.0313, 0.0625, 0.125, 0.25, and 0.5 mg/mL concentrations. The OLE concentration in mg was determined at 280 nm (*y* = 714.02*x*; *R*^2^ = 0.9988), while VER and L4OG were determined at 325 nm (*y* = 2427.6*x*; *R*^2^ = 0.9991, and *y* = 2362.7*x*; *R*^2^ = 0.9998, respectively). All data presented are mean values of two independent experiments (*n* = 2). 

The method was validated by determining linearity and range, accuracy, precision, selectivity, limit of detection (LOD), and limit of quantitation (LOQ). The limits of detection (LODs) and the limits of quantification (LOQs) were estimated from standard solutions. The LODs and LOQs for analyzed phenolics were expressed as mg/mL for OLE, VER, and L4OG. Good linearity with *R*^2^ greater than 0.999 was achieved between 0.02 and 11.2 mg/mL for OLE, 0.03 and 4.0 mg/mL for VER, and 0.03 to 4.7 mg/mL for L4OG. The LODs obtained were 0.002 mg/mL for VER and L4OG, and 0.005 mg/mL for OLE, while the LOQs for OLE, VER, and L4OG were 10.08, 2.8, and 2.5 mg/mL, respectively.

### 2.4. Experimental Design

**Figure 3:**
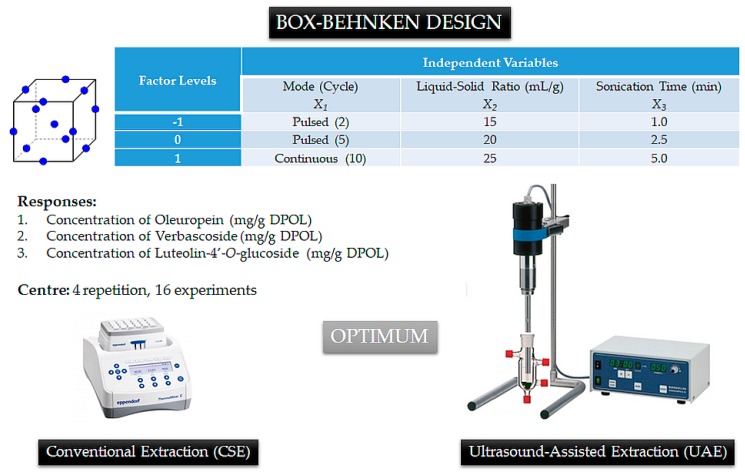
Experimental design with experimental values and coded levels of independent variables used for the Box–Behnken design for ultrasound-assisted extraction (UAE).

In this study, a Box–Behnken design (BBD) of response surface methodology (RSM) was used to identify the optimal levels of independent variables for the dependent variable [17]. To evaluate the pure error, the design consisted of sixteen randomized runs with four replicates at the central values. Independent variables used in the experimental design were ultrasound extraction mode, sonication extraction time, and the L–S ratio (see Figure 3).

As shown in Table 1, the three independent variables were coded at three levels (−1, 0, and +1) and extraction yields of OLE, VER, and L4OG are presented. The parameters obtained from the RSM analysis were substituted into the second-order polynomial model shown in Equation (2).

(2) $$\;Y=\beta_{0\;\;}+\overset{k}{\underset{i\;=\;1}{\sum }}\beta_{i\;\;}X_{i}+\overset{k}{\underset{i\;=\;1}{\sum }}\beta_{\mathrm{ii}\;\;}X_{\mathrm{ii}}^{2}\;+\overset{k-1}{\underset{i}{\sum }}\beta_{\mathrm{ij}\;\;}X_{i\;\;}X_{j}+e,$$

where *Y* is the predicted response (extraction yields of OLE, VER, and L4OG), *X*_i_ (i = 1, 2, 3) are non-coded values of independent variables (ultrasound mode, sonication time, and L–S ratio), *β*_0_ is the constant coefficient; *β*_i_, *β*_ii_, and *β*_ij_ are the linear, quadratic, and cross-product coefficients, respectively, and *e* is error.

In order to have the most suitable set of independent variables, a backward stepwise regression technique was used. This technique, in the beginning, includes all the variables to estimate parameters, before removing variables with a non-significant parameter at a given alpha level. The process continues until there are no significant variables left. RSM modeling enabled deriving of the optimal extraction parameters for the maximization of extraction yields.

### 2.5. Statistical Analysis

All of the experiments were carried out in duplicate and the results are presented as means with standard deviations. The experimental design and multiple linear regression analysis were performed using the Industrial Statistics & Six Sigma module within the data analysis software system TIBCO Software Inc. (2017) and Statistica (data analysis software system), version 13. http://statistica.io. The adequacy of the ANOVA for response surface models for all independent variables without interaction was assessed using the *F*-test and the lack-of-fit test (at a 5% statistical significance level) with an accompanying adjusted *R*^2^ value. The optimal extraction conditions were calculated using the response desirability profiling procedure (at optimal values) for the maximization of the response variable.

## 3. Results and Discussion

In this paper, three important UAE parameters—continuous and pulsed ultrasound operating modes (number of cycles), sonication time, and L–S ratio—were optimized by RSM for three responses (OLE, VER, and L4OG) on the basis of a Box–Behnken design (BBD) for the optimal yields of each, after UAE from DPOL. These three examined responses, except for the large difference in the amount in the plant matrix, also have different structures, solubilities in ethanol, and bioactivities. The results of the BBD showed that the significant variable for all responses was sonification time, while the cycle number was significant for one response (VER) shown through Pareto’s chart of the estimated effects at a 95% confidence level (see Supplementary Materials, Figure S1). This means that only these two variables exhibited significant results.

In a follow-up experiment to this study, an RSM through a BBD with all three independent variables was accomplished to determine the optimal conditions and critical points of these variables.

### 3.1. Modeling of Ultrasound-Assisted Extraction Using RSM

RSM was applied to model the extraction yields of OLE (*Y*_1_), VER (*Y*_2_), and L4OG (*Y*_3_) in dependence of three extraction parameters, namely ultrasound mode (*X*_1_), sonication time (*X*_2_), and the liquid–solid ratio (*X*_3_). The models for each of the responses were analyzed separately before overall processing optimal conditions were determined. Modeling was done using a second-order polynomial, described in Equation (2), and the resulting equations are given as Equations (3)–(5).

(3) $$\;Y_{1}\;=\;10.743\;-\;0.633X_{1}\;+\;0.061X_{1}^{2}\;-\;0.109X_{2}\;+\;0.001X_{2}^{2}\;+\;2.292X_{3}\;-\;0.309X_{3}^{2},\;$$

(4) $$\;Y_{2}\;=\;0.064\;-\;0.015X_{1}\;+\;0.002X_{1}^{2}\;+\;0.010X_{2}\;+\;0.093X_{3}\;-\;0.012X_{3}^{2},\;$$

(5) $$\;Y_{3}\;=\;0.260\;-\;0.025X_{1}\;+\;0.003X_{1}^{2}\;-\;0.006X_{2}\;+\;0.152X_{3}\;-\;0.019X_{3}^{2}.\;$$

As it can be seen from the equations, the modeling process excluded binary interactions (*X*_i_*X*_j_ terms) because they produced significant lack-of-fit values (*p* = 0.040). The adequacy of the models was analyzed using the *F*-test (ANOVA) and the model parameters are presented in Table 2. 

As shown in Table 2, the time of sonication (*X*_3_) significantly affected the yield of OLE for the linear (*p* = 0.028) and quadratic term (*p* = 0.035). The quadratic term of UAE conditions exhibited negative effects, especially in the L–S ratio (*X*_2_; *p* = 0.921). Although the linear term showed higher significance for the time of sonication for the OLE than the quadratic term, some additional effects of the quadratic term existed (*p* = 0.034). The lack of fit for OLE was 6.278 of the *F*-value, implying that the model fits well and has a significant effect on parameters of output response.

A similar finding may be reported for the effects of process parameters on the yield of L4OG. A positive effect was exhibited on the yield of L4OG (*p* = 0.006) in the linear term for the time of sonication (*X*_3_), but other parameters, ultrasound mode (*X*_1_) and the L–S ratio (*X*_2_), showed no significant effect (*p* = 0.079 and *p* = 0.853, respectively). In addition, *R*^2^ (0.856) and pure error *SS* (0.003) for L4OG indicated that the predicted values were in good correlation with the observed values. The lack of fit for L4OG was 1.624 of the *F*-value, indicating that the model fits the output responses well. An additional effect of the quadratic term on the linear term for the time of sonication was also significant, while binary interactions produced no significant lack of fit (*p* = 0.207), meaning the model with two-way linear interactions could also fit well, but with less percentage of chance. In addition, positive effects were obtained in the yield of VER in the linear and quadratic term for the time of sonication (*X*_3_; *p* = 0.002 and *p* = 0.004, respectively) and the number of cycles (*X*_1_; *p* = 0.014 and *p* = 0.002, respectively), and negative effects were obtained for the L–S ratio (*X*_2_; *p* = 0.233 and *p* = 0.381, respectively). The non-significant lack of fit *F*-value for VER of 6.298 means that the model fits well, and had a significant effect on parameters of output response. It should be noted that positive effects for the time of sonication (*X*_3_; *p* = 0.003) and the number of cycles (*X*_1_; *p* = 0.011) were also found in the model with the sum of the linear and quadratic terms. The model with two-way interactions (L by L) was not appropriate because of significant lack of fit (*p* = 0.039). In general, by increasing the L–S ratio, the amounts of phenolic compounds could be decreased as shown in the case of oleuropein. However, in some cases, the increase in yields with the increase in solvent quantity could be contrary to mass transfer principles and lead to higher diffusion. Here, the yields of all dependent variables did not respond to the L–S ratio (15–25 mL/g).

**Figure 4:**
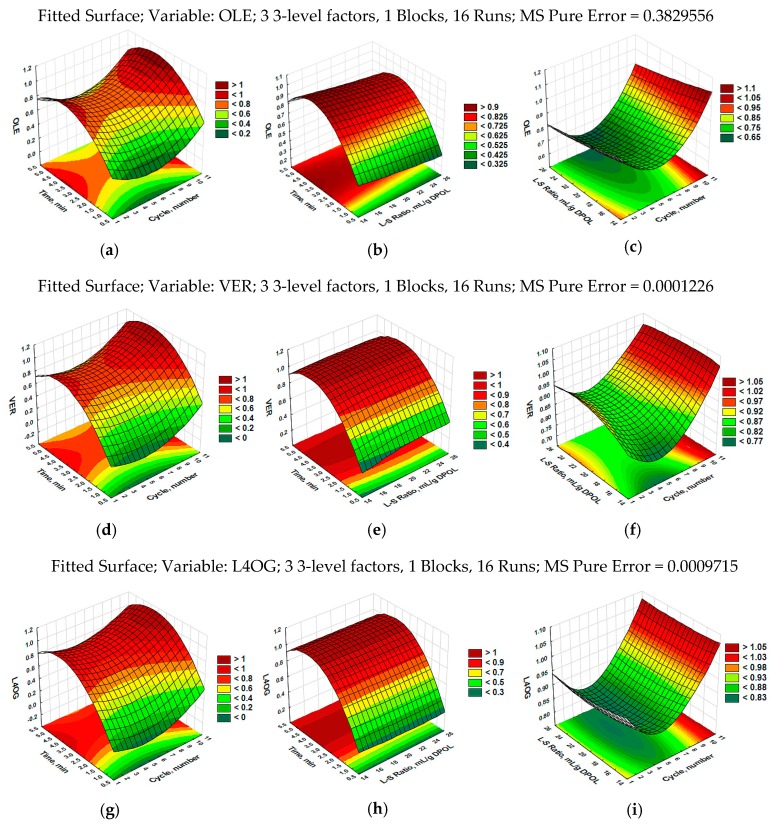
Response surface plots showing the effect of sonication time (*X*_3_) and number of cycles (*X*_1_) on the concentrations of (**a**) oleuropein (OLE), (**d**) verbascoside (VER), and (**g**) luteolin-4′-*O*-glucoside (L4OG); the effect of sonication time (*X*_3_) and liquid–solid (L–S) ratio (*X*_2_) on the concentrations of (**b**) OLE, (**e**) VER, and (**h**) L4OG; the effect of L–S ratio (*X*_2_) and number of cycles (*X*_3_) on the concentrations of (**c**) OLE, (**f**) VER, and (**i**) L4OG in olive leaves as a function of desirable values at optimal conditions and examined parameters of UAE (number of cycles = 10; L–S ratio = 15; extraction time = 4 min) for all dependent variables. All data presented are mean values of two independent experiments (*n* = 2).

The optimal extraction conditions were calculated using the response desirability profiling procedure (at optimal values) for maximization of the response variable. The optimal extraction conditions for OLE, VER, and L4OG were calculated to be: *X*_1_ = 10, *X*_2_ = 15, and *X*_3_ = 4, meaning 10 cycles of ultrasound (100% duty cycle or continuous mode), an L–S ratio of 15 mL/g of DPOL, and an extraction time of 4 min. At these conditions, the maximum extraction yield for OLE was 13.386 mg/g DPOL with 95% confidence intervals (CIs) in the range of 11.964–14.808, for a VER of 0.363 with 95% CIs ranging from 0.338 to 0.389, and for a L4OG of 0.527 with 95% CIs between 0.456 and 0.600 (see Supplementary Materials, Figure S2). Response surface plots were generated to present the effects of UAE processing parameters (number of cycles, L–S ratio, and sonication time) on each variable (OLE, VER, and L4OG) (see Figure 4a–i.). The response surface plots show combined effects of process variables on the responses of OLE, VER, and L4OG at optimal conditions. Negative interactive effects of a combination of sonication time and a number of cycles caused saddle surface, which suggested a further decrease of OLE yield at the longer time of sonication (Figure 4a). Negative effects of the quadratic term on sonication time and the number of cycles started with a decrease in OLE after it reached a maximum (13.386 mg/g DPOL). At the endpoint of sonication time examined, the predicted concentration of OLE was 12.898 mg/g DPOL. The same effects were observed for combinations of sonication time and L–S ratio (Figure 4b) and of L–S ratio and number of cycles (Figure 4c). The predicted concentration for OLE was 12.834 mg/g DPOL at the endpoints of L–S ratio (25 mL/g) and number of cycles (10). Similar effects were observed for VER (Figure 4d–f) and L4OG (Figure 4g–i), meaning that, at the marginal conditions of L–S ratio (25 mL/g) and number of cycles (10), predicted values for VER and L4OG were lower than those at optimal conditions. It is important to emphasize that all variables achieved higher concentrations in the continuous mode of sonication, followed by with a minimal number of cycles (two cycles) in pulse mode.

To verify the reliability of the models, an experiment was performed under the modified optimal conditions at marginal conditions: continuous cavitation cycles (10), L–S ratio 25 (*v/w*), and 5 min for sonication time (for all phenolic compounds investigated). The extraction mean yields for OLE, VER, and L4OG were 11.365 mg/g DPOL, 0.324 mg/g DPOL, and 0.412 mg/g DPOL, respectively, and matched with the predicted values. The relative errors between the predicted and experimental values at marginal conditions were dependent on the examined phenolic compound (OLE 8.63%, VER 11.3%, and L4OG 22.48%). The correlation between the experimental and predicted value computed for extraction recovery of OLE, VER, and L4OG using UAE is shown in the Supplementary Materials, Figure S3. Thus, the regression models obtained using RSM could accurately predict the OLE, VER, and L4OG extraction yields for any combination of cavitation cycles, L–S ratios, and sonication times, despite these compounds being markedly different in their content.

In addition, critical values of UAE extraction parameters were calculated based on the applied model for each of variables: for OLE, number of cycles = 5.207, L–S ratio = 40.606, and extraction time = 3.709 min; for VER, number of cycles = 4.511, L–S ratio = 22.569, and extraction time = 3.859 min; and for L4OG, number of cycles = 4.655, L–S ratio = 21.700, and extraction time = 4.006 min. At these critical values, predicted values for solutions of OLE, VER, and L4OG were 11.136, 0.325, and 0.445, respectively. A comparison of the fitted surface plots for extraction yields of OLE, VER, and L4OG from olive leaves as a function of critical and optimal conditions are presented in the Supplementary Materials, Figure S4. Almost all critical values obtained from the model were within the examined ranges of cycle number (2–10), L–S ratio (15–25), and time of sonication (1–5 min), except L–S ratio for OLE. In addition, predicted values for solutions of OLE, VER, and L4OG were lesser than, equal to, and higher than values obtained from the model at optimal conditions, respectively. 

Considering the different structures, hydrophilicities (XLogP3 values for OLE, VER, and L4OG are −0.4, −0.5, and 0.5, respectively), and particularly the different contents of these phenolic compounds in the plant matrix (OLE contributes greater than 90% of phenolic compounds in olive leaves), optimal conditions were found and yields of OLE, VER, and L4OG were satisfactory. Due to limited reports, the data obtained in this study could not be fully compared with other authors. 

Ilbay et al. [21] demonstrated an artificial neural network (ANN) model and RSM with five parameters (pH, extraction time, extraction temperature, and solid/solvent ratio) and one response (total phenolic content, TPC) in order to obtain optimal conditions for the evaluation of UAE of olive leaf phenolic compounds [21]. Using RSM and BBD, optimal values were found at 56.17 mg GAE/g of DPOL at a pH value of 3.52, a temperature of 59.87 °C, an extraction time of 59.57 min, and an L–S ratio of 39.56. The linear, square, and interaction coefficients for all parameters were statistically significant for all parameters, except for the interaction of pH and extraction time. 

The optimization of UAE extraction parameters (solvent concentration, S–L ratio, and extraction time) was performed using RSM to obtain optimal processing conditions [19]. Using the second-order polynomial model in the description of the experimental data and the predicted responses, the optimal process conditions were determined as 201.2158 mg/g of DPOL extract, 25.0626 mg GAE/g of DPOL, and 95.5610% DPPH (for an L–S ratio of 20 mL/g of DPOL, 60 min extraction time, and 50% ethanol).

Japon-Lujan et al. [22] proposed a multivariate methodology to optimize the extraction process a continuous UAE of olive biophenols (OBPs) from olive leaves. Under the optimal working conditions, complete extraction of the target analytes (oleuropein, verbascoside, apigenin-7-glucoside, and luteolin-7-glucoside) was done in 25 min (LODs were 11.04, 2.68, 1.49, and 3.91 mg/kg, respectively). The amount of OBPs in the extract decreased at higher pH (at 12). Apigenin-7-glucoside was lower by 27%, OLE and luteolin-7-glucoside by 35%, and VER by 40%.

Our study showed the successful use of experimental design (BBD) and RSM in the optimization of UAE to obtain a maximal concentration of OLE, VER, and L4OG from olive leaves. We found that the dependent variables (OLE, VER, and L4OG) did not yield significant results within the limited range of the L–S ratio that was used (15–25 mL/g). Furthermore, we assumed that a longer time duration could have a potential influence on (with possible degradation of) the examined phenolic compounds; thus, the sonication treatment was shorter during extraction. Therefore, the sonication time was determined over 4 min under optimal conditions (in the continuous mode or under 10 cycles; the L–S ratio was 15), which was considerably shorter than in previously mentioned studies.

### 3.2. Comparison of Conventional (CSE) and Ultrasound-Assisted Extraction (UAE) Methods

The comparison between the extraction methods (UAE and CSE) demonstrated higher yields of OLE, VER, and L4OG in the continuous mode of UAE at a temperature 60 °C and an L–S ratio of 15 (*v/w*) than in the CSE. An L–S ratio of 15 was chosen because of the previously obtained optimal response of OLE under these conditions. 

As expected, the yields of all examined phenolic compounds were higher in UAE than in the CSE at all time points set due to ultrasonic breaking down of plant cell walls and easier penetration of solvent into the matrix. 

The UAE increased the yields of OLE by 32.6, 30.3, and 32.9% in set time points (1, 2.5, and 5 min), the yields of VER showed increases of 41.8, 41.3, and 40.6%, while L4OG increased by 47.5, 44.9, and 42.3% in relation to CSE under the same extraction conditions (same temperature, L–S ratio, and extraction times; see Supplementary Materials, Table S1). This means that the extended time of extraction, in these extraction conditions, did not affect the increased recovery of OLE, VER, and L4OG.

Japon-Lujan et al. [22] compared the efficacy of UAE with conventional extraction (CSE) to extract olive biophenols (OBPs) from olive leaves. The same conditions were used for UAE and CSE (an L–S ratio of 8 mL/g, 59% aqueous ethanol, and a temperature of 40 °C). Different extraction times were tested for CSE and UAE. The same percentages of OBPs (80 and 100%, respectively) were obtained for 10 and 24 h at CSE and for 16 and 25 min at UAE. However, there are no data describing how much more effective UAE was than CSE for the same time of extraction.

Determination of the kinetics of UAE and CSE indicated that phenolic extraction was faster in UAE than in CSE [23]. However, both CSE and UAE increased the TPC significantly, as well as the antioxidant capacity and the OLE content at a higher temperature (50 °C). The OLE content reached 6.57 ± 0.18 g/100 g DPOL in the first minute in UAE experiments (or approximately 88%).

These results should certainly be supplemented with the origin of the olive leaves, the season of harvesting, the particle size of the powder after the milling of olive leaves, etc. The time of exposure to ultrasound was important in all mentioned studies including this study. In addition, the difference in ultrasound exposure in some studies could be due to the particle size of the plant materials. In our case, a longer exposure of small particles to ultrasound had a consequential reduction in cavitation due to sonoporation and formation of a deposit on the probe of the transducer. We assumed the reason could be the degradation of phenolic compounds due to the elongation of sonication time. Finally, the proposed model could be a reliable tool in the UAE processing studies, as it was able to predict and describe the behaviour of the dependent variables. Further research will focus on the particle size at extraction time, as well as novel extraction technologies of high-added-value compounds from plant materials. 

## 4. Conclusions

This work studied the effects of the ultrasound mode (pulsed and continuous), the L–S ratio, and the sonication time on the UAE extraction of olive leaves. After UAE was conducted, OLE, VER, and L4OG were analyzed and quantified using UPHLC-DAD. RSM using the BBD was performed for the optimization of experimental conditions for OLE, VER, and L4OG extracted from olive leaves.

The optimal extraction conditions for all examined phenolics were as follows: number of cycles (*X*_1_) = 10, liquid–solid ratio (*X*_2_) = 15 mL/g of dry powdered olive leaves (DPOL), and sonication time (*X*_3_) = 4 min. Under these optimal conditions, the concentration of OLE after UAE was 13.386 mg/g DPOL with 95% CIs from 11.964–14.808, the concentration of VER extraction was 0.363 mg/g DPOL with a 95% CIs of 0.338–0.389, and the concentration of L4OG extraction was 0.527 mg/g DPOL with 95% CIs ranging from 0.456–0.600.

Verification of experiments was carried out under modified optimal conditions at marginal conditions under the continuous ultrasonic operating mode as follows: number of cycles (*X*_1_) = 10, liquid–solid ratio (*X*_2_) = 25 mL/g DPOL, and sonication time (*X*_3_) = 5 min. The extraction yields for OLE, VER, and L4OG were 11.365 mg/g DPOL, 0.324 mg/g DPOL, and 0.412 mg/g DPOL, respectively, which was in good agreement with the predicted values. The relative errors between the predicted and experimental values depended on phenolic compound type, and they were 8.63% for OLE, 11.3% for VER, and 22.48% for L4OG. These regression models can accurately predict the OLE, VER, and L4OG extraction yields for any combination of cavitation cycles, L–S ratios, and sonication times.

In comparison with CSE, UAE improved the yields of OLE (32.6%, 30.3%, and 32.9%, respectively), VER (41.8%, 41.3%, and 40.6%, respectively), and L4OG (47.5%, 44.9%, and 42.3%, respectively) at a temperature of 60 °C, an L–S ratio of 15 (*v/w*), and a continuous mode of UAE.

## Figures and Tables

**Table 1:** Experimental design in coded and uncoded form, and values of experimental data (yields of oleuropein (OLE), verbascoside (VER), and luteolin-4′-*O*-glucoside (L4OG)) using the Box–Behnken design (BBD) matrix. DPOL—dry powdered olive leaves.

Runs	Extraction Conditions						Analytical Results (mg/g DPOL)		
	Uncoded Values			Coded Values					
	Cycle *X*_1_	L–S Ratio ^a^ *X*_2_	Time *X*_3_				Oleuropein	Verbascoside	Luteolin-4′-*O*-Glucoside
1	5	20	2.5	0	0	0	11.284 ± 0.14	0.306 ± 0.00	0.432 ± 0.01
2	10	25	2.5	1	1	0	12.261 ± 0.05	0.351 ± 0.00	0.484 ± 0.00
3	5	20	2.5	0	0	0	11.181 ± 0.00	0.298 ± 0.00	0.422 ± 0.00
4	10	15	2.5	1	−1	0	13.517 ± 0.10	0.349 ± 0.01	0.493 ± 0.01
5	5	25	5	0	1	1	11.792 ± 0.04	0.322 ± 0.01	0.448 ± 0.01
6	10	20	5	1	0	1	12.120 ± 0.11	0.349 ± 0.00	0.490 ± 0.01
7	5	20	2.5	0	0	0	10.523 ± 0.01	0.288 ± 0.00	0.393 ± 0.02
8	5	25	1	0	1	−1	8.995 ± 1.56	0.220 ± 0.04	0.264 ± 0.05
9	5	15	1	0	−1	−1	7.789 ± 0.00	0.178 ± 0.00	0.234 ± 0.01
10	5	20	2.5	0	0	0	12.034 ± 0.00	0.314 ± 0.00	0.363 ± 0.00
11	2	20	5	−1	0	1	10.122 ± 0.03	0.288 ± 0.00	0.402 ± 0.01
12	2	15	2.5	−1	−1	0	12.271 ± 0.18	0.302 ± 0.01	0.427 ± 0.01
13	2	25	2.5	−1	1	0	11.144 ± 0.14	0.305 ± 0.00	0.408 ± 0.01
14	2	20	1	−1	0	−1	12.165 ± 0.11	0.271 ± 0.00	0.349 ± 0.00
15	5	15	5	0	−1	1	12.822 ± 2.51	0.323 ± 0.07	0.469 ± 0.09
16	10	20	1	1	0	−1	10.894 ± 0.00	0.281 ± 0.00	0.350 ± 0.00

^a^ L–S ratio—Liquid-solid ratio (mL/g DPOL).

**Table 2:** ANOVA for response surface models (RSM) for all independent variables (model: no interactions, real values of independent variables).

Factor	Oleuropein (*Y*_1_)					Verbascoside (*Y*_2_)					Luteolin-4′-*O*-Glucoside (*Y*_3_)				
	*SS*	*dF*	*MS*	*F*	*P*	*SS*	*dF*	*MS*	*F*	*P*	*SS*	*dF*	*MS*	*F*	*P*
Model	31.135	15				0.031	15				0.086	15	<0.001		
Linear (L)															
*X*_1_	1.193	1	1.193	3.114	0.176	0.003	1	0.003	27.143	0.014 *	0.007	1	0.007	6.864	0.079
*X*_2_	0.610	1	0.610	1.591	0.296	<0.001	1	<0.001	2.215	0.233	<0.001	1	<0.001	0.040	0.853
*X*_3_	6.149	1	6.149	16.057	0.028 *	0.014	1	0.014	113.11	0.002 **	0.047	1	0.047	48.129	0.006 *
Quadratic (Q)															
*X*_1_^2^	3.230	1	3.230	8.436	0.062	0.003	1	0.003	20.882	0.002 **	0.006	1	0.006	6.471	0.084
*X*_2_^2^	0.004	1	0.004	0.012	0.921	<0.001	1	<0.001	1.049	0.381	<0.001	1	<0.001	0.044	0.848
*X*_3_^2^	5.208	1	5.208	13.600	0.035 *	0.008	1	0.008	65.287	0.004 **	0.002	1	0.002	20.251	0.020 *
Sum of L and Q															
*X*_1_ *+ X*_1_^2^	5.266	2	2.633	6.875	0.076	0.007	2	0.004	29.037	0.011 *	0.016	2	0.008	8.073	0.062
*X*_2_ *+ X*_2_^2^	0.614	2	0.307	0.802	0.526	<0.001	2	<0.001	1.632	0.331	<0.001	2	<0.001	0.042	0.959
*X*_3_ *+ X*_2_^2^	9.680	2	4.840	12.639	0.034 *	0.019	2	0.009	76.560	0.003 **	0.058	2	0.029	29.654	0.011 *
Lack of fit	14.426	6	2.404	6.278	0.080	0.005	6	<0.001	6.298	0.080	0.009	6	0.001	1.624	0.370
Pure error	1.148	3	0.383			<0.001	3	<0.001			0.003	3	0.001		
Total SS	31.135	15				0.031	15				0.086	15			
*R* ^2^	0.500					0.840					0.856				
*Adj R* ^2^	0.166					0.734					0.759				

*X*_1_, *X*_2_, and *X*_3_ represent the number of cycles, liquid-to-solid ratio, and sonication time, respectively; *SS*, *dF* and *MS* represent the sum of squares, degrees of freedom, and mean square, respectively. * Significant at *p* < 0.05; ** significant at *p* < 0.005.

## Supplementary Materials

The following are available online at http://www.mdpi.com/2304-8158/7/9/149/s1, Figure S1: Pareto chart obtained from Box–Behnken design in the optimization of (a) OLE, (b) VER, and (c) L4OG after of UAE. Figure S2: Profiles for predicted values and desirability in the optimization of OLE, VER, and L4OG after UAE. Figure S3: The correlation between the experimentally obtained values of extraction yield of OLE, VER, and L4OG versus the calculated values using the model equation. Figure S4: Fitted surface plots for the extraction yields of OLE, VER, and L4OG in olive leaves at calculated critical conditions (a1–a3) OLE, (c1–c3) VER, and (e1–e3) L4OG; and at obtained optimal conditions for all dependent variables (b1–b3) OLE, (d1–d3) VER, and (f1–f3) L4OG. All data presented are the means of two independent experiments (*n* = 2). Table S1: Comparison between UAE and CSE.
