# Supplementary material for: Optimization of Ultrasonic-Assisted Extraction of Major Phenolic Compounds from Olive Leaves (Olea europaea L.) Using Response Surface Methodology
Source: Foods. 2018 Sep 6;7(9):149. doi: 10.3390/foods7090149 (PMC6165173; doi:10.3390/foods7090149)
Supplement: Supplementary file 1 [file foods-07-00149-s001.pdf]

Supplementary Materials:

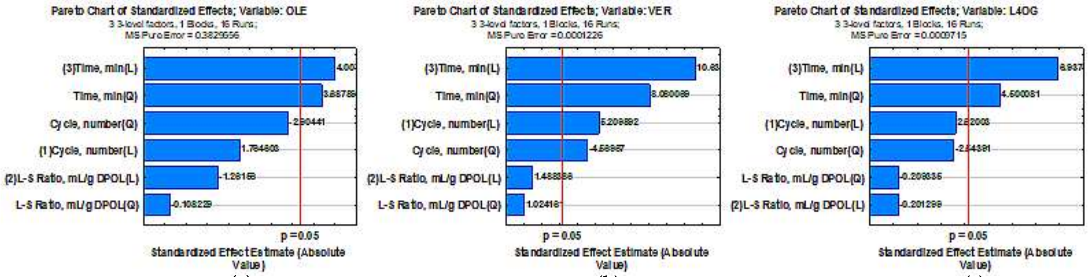

(a) (b) (c)

Figure S1. Pareto chart obtained from Box–Behnken design in the optimization of (**a**) OLE, (**b**) VER, and (**c**) L4OG after of UAE.

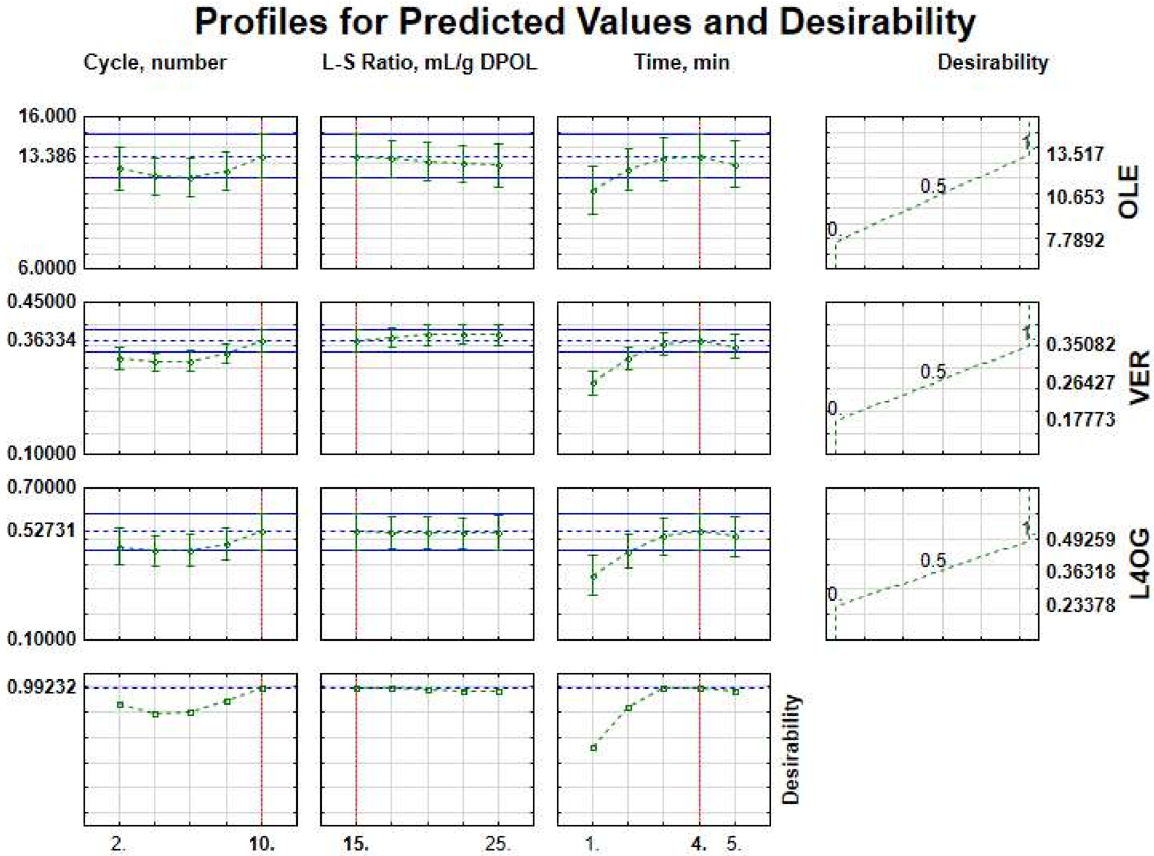

Figure S2. Profiles for predicted values and desirability in the optimization of OLE, VER, and L4OG after UAE.

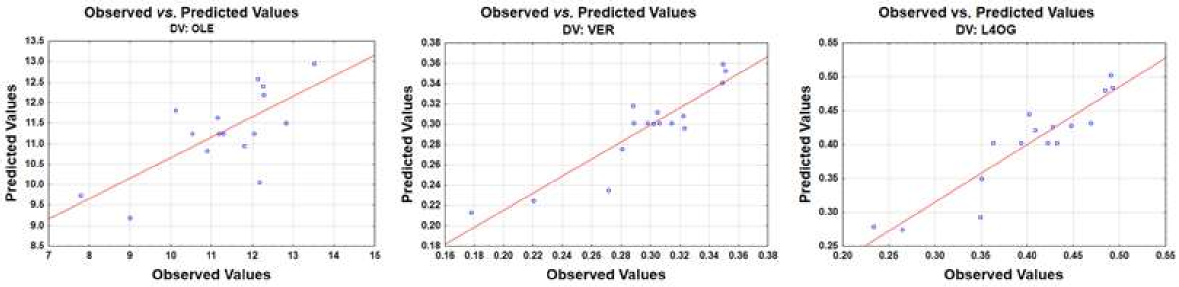

Figure S3. The correlation between the experimentally obtained values of extraction yield of OLE, VER, and L4OG versus the calculated values using the model equation.

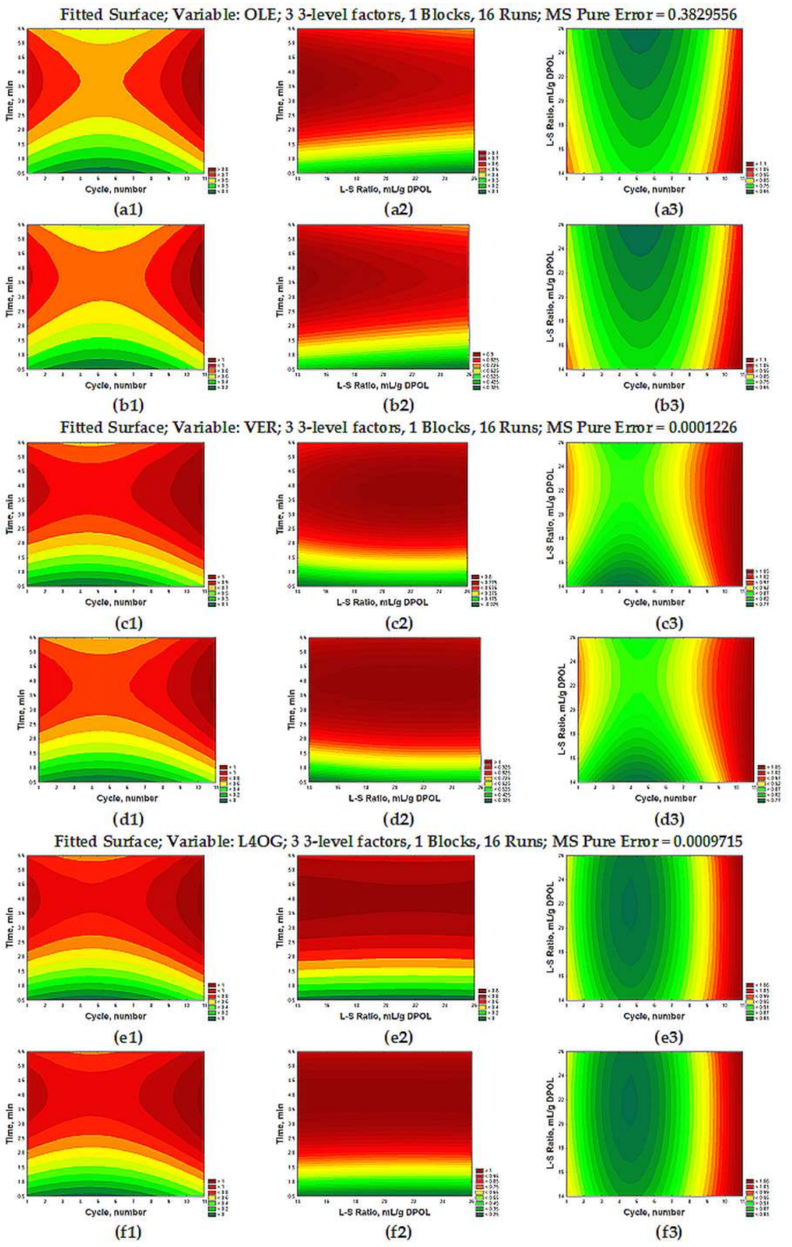

Figure S4. Fitted surface plots for the extraction yields of OLE, VER, and L4OG in olive leaves at calculated critical conditions (**a1**–**a3**) OLE, (**c1**–**c3**) VER, and (**e1**–**e3**) L4OG; and at obtained optimum conditions for all dependent variables (**b1**–**b3**) OLE, (**d1**–**d3**) VER, and (**f1**–**f3**) L4OG. All data presented are the means of two independent experiments (n = 2).

Table S1. Comparison between UAE and CSE.

Variable	CSE Time, min			UAE Time, min		
	1.0	2.5	5.0	1.0	2.5	5.0
OLE, mg/g DPOL	7.97	9.42	9.92	11.81	13.52	14.78
VER, mg/g DPOL	0.16	0.20	0.23	0.28	0.35	0.39
L4OG, mg/g DPOL	0.20	0.27	0.32	0.39	0.49	0.56
UAE-CSE difference, % [b]						
OLE				32.6	30.3	32.9
VER				41.8	41.3	40.6
L4OG				47.5	44.9	42.3

UAE and CSE was carried out with 80% aqueous ethanol at 60 °C, and an L-S ratio of 15 mL/g of DPOL.

All presented data are mean values of two independent experiments (n = 2). The difference between UAE and CSE was calculated as:

[b] $$Difference\ between\ UAE\ and\ CSE\ (\%) = \left(1 - \frac{Concentration\ of\ Phenolic\ compound\ (CSE)}{Concentration\ of\ Phenolic\ compound\ (UAE)}\right) \times 100$$
